# Psychosocial Problems among Psychiatric Nurses for Caring Patients with Mental Disorders during the COVID-19 Pandemic

**DOI:** 10.1155/2023/3689759

**Published:** 2023-06-24

**Authors:** Ibrahim Rahmat, Fajar Pawestri, Ragil Aji Saputro, Setiyati Widianingrum, Triana Hanifah

**Affiliations:** ^1^Department of Mental Health and Community, School of Nursing, Faculty of Medicine, Public Health, and Nursing, Universitas Gadjah Mada, Yogyakarta, Indonesia; ^2^School of Nursing, Faculty of Medicine, Public Health, and Nursing, Universitas Gadjah Mada, Yogyakarta, Indonesia

## Abstract

COVID-19 has a negative effect on the psychological well-being of psychiatric nurses. Thus, examining the psychosocial response of nurses is important for preventing more serious mental health problems and disruption of the quality of nursing care. This study aimed to evaluate the psychosocial problems of nurses who provided nursing care to patients with mental health disorders during the COVID-19 pandemic. A quantitative study with a cross-sectional design was conducted. The 101 nurses at Central Mental Health Hospital who provide nursing care to patients with mental health disorders were recruited through consecutive sampling. The instruments used were the demographic questionnaire, the Expanded Nursing Stress Scale, Zung Self-Rating Anxiety Scale, and The Connor–Davidson Resilience Scale 25. Univariate and bivariate analyses were used to process the data. The mean score of 45.1 (±24.3) was obtained for the ENSS; around 97% of nurses have a work stress score below the average, 4.95% have mild-moderate anxiety, and 28.7% have a low level of resilience. Work stress and contact frequency, work stress and gender, anxiety and contact frequency, as well as resilience and contact frequency all correlated significantly (*p* value *<*0.05). The Pearson test showed a significant positive correlation between work stress and anxiety (*p*: 0.002, *r*: 0.299). However, there was no significant correlation between anxiety and resilience (*p*: 0.643, *r*: 0.47), nor between work stress and resilience (*p*: 0.643; *r*: 0.47). Psychosocial disorders that psychiatric nurses face include occupational stress, mild-moderate anxiety, and low resilience. The government can create specific infection control guidelines for the mental health setting, and hospital management or ward leaders can also provide support to psychiatric nurses to increase resilience in reducing psychosocial problems.

## 1. Introduction

The COVID-19 pandemic developed into a global health crisis with a considerable increase in cases and a high death rate worldwide. It is a challenge for health workers, especially nurses. Along with these issues, nurses are prone to internal problems relating to severe workloads, insomnia, conflicts among health workers, moral concern, and professional dilemmas [1, 2]. COVID-19 does not only influence physical health problems but also on psychosocial health. The impact of the COVID-19 pandemic on psychosocial aspects includes symptoms of stress, anxiety, depression, and posttraumatic stress disorder [3]. Psychiatric nurses frequently lack knowledge about infectious disease management and must process a large amount of information about COVID-19 in a short period of time, while also having fears of transmitting the disease to patients, limited personal protective equipment, and conflicts among health workers, which causes psychosocial disorders to nurses [4, 5].

Several research articles have proven that nurses experience high levels of stress and anxiety during COVID-19. Anxiety was assessed in 89.7% of Iranian nurses, as was stress in 54.9% of them [6]. Huang et al. [7] found that out of 1,257 health workers caring for COVID-19 patients in 34 hospitals in China, 50% experienced depressive symptoms and 45% ran into anxiety. Nurses must be resilient to deal with challenging conditions that are riddled with problems and challenges. Since resilience is one of the factors that protect nurses from rising when faced with challenges, emotional tiredness, and job dissatisfaction, it allows nurses to retain healthy and stable psychological functions and adapt to the work environment [8]. Research conducted on 824 nurses in Korea reported that nurses with depression experienced lower resilience and were prone to experiencing higher work stress [9].

Preliminary research was undertaken on psychiatric nurses at Central Mental Health Hospital. Researchers conducted interviews with three psychiatric nurses about psychosocial problems during COVID-19. Based on interviews, they encounter a variety of psychosocial disorders, such as difficulty conditioning patients—for instance, those who perform risky behavior like careless spitting—difficulties communicating with patients as a result of the use of personal protective equipment (PPE), and anxiety when other patients or other nurses are confirmed to be positive for COVID-19. Research on psychological aspects has not been widely conducted concerning work stress, anxiety, and the resilience of psychiatric nurses. Therefore, this study aims to provide information about the psychosocial problems of psychiatric nurses who provided nursing care to patients with mental problems during the COVID-19 pandemic.

## 2. Methods

### 2.1. Research Design

This research is a descriptive one that uses a cross-sectional design and a quantitative approach. This research was conducted in June 2022.

### 2.2. Subjects

The population in this study was psychiatric nurses at the Central Mental Health Hospital in Yogyakarta, Indonesia. Psychiatric nurses are defined as nurses who specialize in psychiatric nursing, caring for patients with mental and behavioral disorders holistically, not only providing physical care but also socializing and communicating with patients to improve their physical and psychosocial well-being. They also provide comprehensive care to individuals, families, groups, and communities. Meanwhile, Bowers et al. [10] define psychiatric nurses as a group of nurses who interact face-to-face with patients in inpatients settings who experience stress and/or frustration at applicable restrictions, which include actions or procedures to maintain security, seclusion, restraint, and enforced medication. Consecutive sampling was used as the sampling method. The inclusion criteria were that the nurses had to have at least a Diploma in Nursing and be willing to participate in the survey. They also had to have provided nursing treatment to patients with mental problems at Mental Health Hospital during the COVID-19 pandemic. Nurses on leave from their employment were excluded from the sample. The total population of nurses who provide nursing care to patients with mental disorders is 122. The researcher determined the sample size using the Slovin formula with a 50% margin of error (*e*: 0.05), so that the minimum sample size was 94 nurses. A total of 101 nurses were accepted as participants in this study.

### 2.3. Instruments

The Expanded Nursing Stress Scale (ENSS) is an instrument used to measure the work stress of nurses. There are 27 questions on it, and the results are divided into scores above and below average. The ENSS instrument has a Cronbach's alpha of 0.956 with a reliability coefficient 0.3181. The Zung Self-Rating Anxiety Scale (Z-SAS) measures anxiety levels which are divided into normal/not anxious, mild to moderate, severe, and panic [11]. At Yogyakarta General Hospital, 75 nurses participated in a construct validity test using the Z-SAS instrument. The results were *r count* >0.3 and Cronbach's alpha 0.658.

The Connor–Davidson Resilience Scale 25 (CD-RISC 25), which contains 25 statements to measure participant resilience. The data were categorized into low, medium, and high resilience levels [12]. The researcher conducted a construct validity test at Yogyakarta General Hospital and Surakarta Regional Mental Hospital and obtained a reliability coefficient of 0.281–0.766 and Cronbach's alpha 0.917.

### 2.4. Data Collection

This research was conducted at the Central Mental Health Hospital in Yogyakarta for psychiatric nurses. Questionnaires were given in printed form and digital form via links which contained data on participant characteristics and research instruments.

### 2.5. Data Analysis

Participants' answers were analyzed using univariate and bivariate analyses. The independent *T*-Test, Mann–Whitney, and Kruskal–Wallis tests were used to compare the work stress levels of the participants' using the ENSS instrument. The Mann–Whitney test was utilized to examine data from the Z-SAS, while the chi-Square and Fisher tests were used to examine the CD-RISC25 instrument. The correlation between work stress and anxiety as well as the correlation between work stress and resilience were examined using the Pearson product-moment correlation test. Furthermore, the correlation between anxiety and resilience was examined using Spearman's rank correlation test.

### 2.6. Ethical Considerations

This research was approved by the Ethics Commission of FK-KMK UGM with number KE-FK-0044-EC-2022.

## 3. Results

### 3.1. Sample Characteristics

A total of 101 nurses participated in this study. A total of 4 questionnaires were given to all participants. The instruments we provided included demographic data, ENSS, Z-SAS, and CD-RISC 25. The researchers gave questionnaires and explained how to fill them out to all participants and they agreed to consent and completed all questionnaires.  Table 1 shows that characteristics are dominated by those between the ages of 41–65 (56.4%). Most of the nurses were female (67.3%) and married (96%). The proportion of nurses who worked more than 15 years is 71.3%, and most participants contacted patients >5 times (57.4%) each shift. Additional demographic characteristics at baseline of the study sample are shown in Table 1.

### 3.2. Level of Work Stress, Anxiety, and Resilience

Based on the data, the work stress score was 45.1 (SD = 24.3). The prevalence of psychiatric nurses who experienced work stress with a lower than average score is 97% and only 3% have a score higher than average. Most nurses do not experience anxiety or have normal anxiety (95.05%), and only 4.95% of nurses experience mild anxiety. Meanwhile, 51.5% of nurses have moderate resilience, 28.7% still have poor resilience, and just 19.8% have high resilience (see Table 2).

### 3.3. Differences between Work Stress, Anxiety, and Resilience with Demographics

Table 3 shows there were significant results for gender characteristics (*p*=0.013) and contact frequency (*p*=0.002) on the work stress variable. However, there was no significant difference in the correlation between work stress and age, marital status, level of education, and the length of work experience. Nurses with higher stress scores were aged 19–40 (45.77 ± 22.84), married (45.54 ± 23.22), worked ≤15 years (47.9 ± 23, 82), female, and had contact with the patient more than five times.

### 3.4. The Domains of ENSS, Z-SAS, and CD-RISC 25

The ENSS is reported to be made up of nine domains (see Table 4). The highest average scores were uncertainty concerning treatment (25.91 ± 15.33), followed by problems with patients (24.07 ± 10.77). In the Z-SAS instrument, the domain of affective symptoms has the highest median value. The hardiness aspect has the highest median resilience score of all other factors.

### 3.5. Correlation between Work Stress, Anxiety, and Resilience

Pearson product-moment test results showed a significant association between work stress and anxiety (*p* value <0.05). The *r* value of 0.299 indicates a positive relationship between work stress and anxiety (see Table 5). Spearman's rank test stated that there was no significant correlation between anxiety and resilience (*p* value <0.643, *r* 0.47). Work stress and resilience had no significant correlation (*p* value: 0.643, *r*: 0.76).

## 4. Discussion

### 4.1. Levels of Work Stress, Anxiety, and Resilience in Nurses

In this current study, most psychiatric nurses during the COVID-19 pandemic had work stress lower than the average score (<45.1). Research by Mo et al. [13] showed that 22.2% of nurses had scores >50, with the average work stress experienced by nurses of 39.91 points. Meanwhile, Baye et al. [14] stated that 90% of nurses in psychiatric wards are less likely to experience work stress than nurses working in outpatient care. The low level of work stress for health workers during the pandemic proves that they show professional devotion and altruism [15]. In line with the study by Ko et al. [16] in Taiwan, many nursing staff were dedicated to their work under the professional responsibility of caring for patients, even volunteering to be involved on the front lines during the SARS pandemic. However, these findings contradict the findings of Alnazly et al. [17], who discovered that 35% of nurses who handled patients with COVID-19 suffered severe stress.

In some mental health hospitals, inpatient psychiatric patients are placed in wards with many other patients. This condition makes social distancing hard to implement in inpatient wards because the rooms occupied by several patients are cramped and crowded. This suggests maintaining a social distance of 2 meters is less applicable and impractical for proper infection control in inpatient wards [18]. Furthermore, nurses tolerate aggressive behavior exhibited by patients, such as breaking protective glasses, tearing masks, and even spitting. The lack of preparedness in dealing with the COVID-19 pandemic has become a major source of stress for nurses. Foye et al. [18] stated that the use of PPE is a real challenge in emergencies; for example, the act of restraining a patient, where nurses may not have time to wear PPE or even PPE that pose a risk to nurses. So, devotion to the use of PPE guidelines as infection control is not maximally done. There is a need for further attention, guidance, and support for nursing staff in trying to manage the challenges of using PPE on the wards of patients at risk of suicide and keeping patients and staff safe. Many of them also have conflicts with professional ideals, such as feeling sad for not being able to treat patients as well as nurses in the ICU and dread spreading the virus to family members [4]. The stressors above significantly increase work stress for nurses.

Normal anxiety was assessed in 95.05% of participants, and none of them experienced severe anxiety or panic. Similar to the study of Neupane et al. [19] which found that 88.4% of nurses experienced normal anxiety and another 10.5% reported mild to moderate levels of anxiety. A survey in China revealed that less than half of healthcare personnel, notably female nurses caring for patients, exhibit signs of anxiety [2]. The findings of this study contrast with those of a meta-analysis performed by Al Maqbali et al. [20]; which revealed that 37% of nurses reported having anxiety across all 93 journals examined.

After all, the COVID-19 pandemic has been going on for more than two years. The Indonesian Ministry of Health set guidelines for the prevention and control of COVID-19 infection in 2020, whereby all health services are no exception to implementing these regulations including the use of PPE, infectious zones, flow of services, and so on. However, these guidelines often confuse nurses because they are hard to apply in psychiatric hospitals where the patients there have different criteria from other general patients. Meanwhile, the first COVID-19 vaccine for health workers began in early 2021. Until these data were collected, psychiatric nurses had received three doses of the vaccine. PCR swabs are carried out if the nurse shows symptoms or has contact with a COVID-19 patient. The Central Mental Health Hospital already follows the guidelines, which helps them reduce the level of anxiety among the nurses there. This supports the results of other studies by Hennein et al. [21] and Yeung et al. [22] that the absence of COVID-19 control guidelines in hospitals correlates with higher symptoms of anxiety.

The resilience level of nurses is mostly in the moderate category. Moderate resilience means that nurses have demonstrated a positive adaptation function in dealing with adversity during a pandemic. Nurses with higher resilience may be able to adapt to the difficulties of working during a pandemic and are less at risk of experiencing symptoms of trauma and disruption at work [23]. However, some nurses in this study lacked resilience (28.7%). Low resilience may be a sign that one needs to develop coping and adaptive skills to deal with adversity [12].

### 4.2. Differences in Work Stress, Anxiety, and Resilience in Nurses Based on Demographic Characteristics

Significant results were found regarding the difference between work stress and gender. Women have higher work stress scores than men. This is in line with the findings of Tsegaw et al. [24] in their research which revealed that one of the factors is the higher role of women in the family and society. Therefore, there is a significant difference in contact frequency and work stress. Nurses who interact with patients more than five times each shift typically experience significant levels of stress. In this study, it was found that it is common for nurses to interact with patients more than ten times in a shift. According to Kisely et al. [25], the more frequently nurses contact patients, the more vulnerable they are to experience stress disorders, especially during a pandemic because nurses contact confirmed patients more often.

Participants aged less than 40 years showed a higher average stress score. Senior nurses can adopt healthy stress-coping strategies because they are more aware of the possibility of a pandemic and are more responsive to patients [26]. Kuo et al. [27] in their research found no relationship between work stress and education level. They continued by explaining that this occurred because the pandemic produced new issues that had never been encountered before, causing nurses with high and low educational levels to experience the same psychological effects.

Anxiety has a significant relationship with contact frequency. Tang et al. [28] reported that the correlation between contact frequency and traumatic stress experiences can make nurses more anxious. In this study, the more often nurses contacted patients, the lower the anxiety score. Higher anxiety scores occur in the age group under 40 years. This study is in contrast to the findings of Wang et al. [29], which stated that at age above 40, anxiety is 0.4 times higher than at age below. This indicates that as experience increases, the ability to regulate psychology will increase.

In this study, work experience did not significantly affect resilience either, but groups with >15 years of work experience showed higher levels of resilience. The results of this study are different from studies by Foster et al. [30] on mental health nurses in Australia which show a significant relationship between resilience and length of service. According to research by Wu et al. [31], nurses with more than 15 years of experience were more resilient. About 31% of the nurses in this study with fewer than 15 years of experience reported having low resilience. Nurses struggle to handle pressure, the risk of infection, the increased workload, and the risk of mental health disorders since they are less skilled at their professions [32].

Nurses who come with a diploma degree have lower resilience levels than those with a bachelor's degree. The difference between a diploma degree and a nursing bachelor's degree in Indonesia is that a diploma degree, also called vocational education, is more focused on practice and takes three years. Meanwhile, a bachelor's in nursing takes four years, focusing more on understanding theory and critical thinking. Also, nursing bachelors who will work in hospitals must go through clinical stages as an implementation of theory and practice in the field. The findings of Afshari et al. [33] on 387 nurses diverge from this result in that resilience and education level differed significantly. According to Ang et al. [34] and Jamebozorgi et al. [35], nurses have greater resilience with more education. The higher nurses' education level, the more efficiently they use social resources, such as learning to access resources and increasing their use, resulting in greater resilience. As stated by Afshari et al. [33], training is required for nurses to strengthen their skills and knowledge in dealing with the COVID-19 pandemic.

The previous study stated that males generally have a higher level of resilience than females [36, 37]. The same finding is found in this study that females have lower resilience. Male and female nurses differ in their resilience due to variances in coping methods in a cultural setting where women are responsible for the home and job while men are more focused as nurses [36].

### 4.3. The Domain of Work Stress, Anxiety, and Nurse Resilience Instruments

In this study, the first rank of work stress in the ENSS domain is uncertainty concerning therapy. The doctor's absence during a medical emergency, exposure to workplace safety and health, insufficient information from the doctor regarding the patient's medical state, and insufficient time to perform nursing assignments are all factors that could have an impact. The second is the problem with the patient domain. Nurses' work stress is increased by the patient's unreasonable requests and inappropriate behavior, which is typically verbal [5]. There are many psychiatric wards where patients are free to move around and interact with other patients. Nurses had difficulty isolating patients with behavioral deregulation from symptoms of mania and active psychosis during the COVID-19 pandemic. They also have difficulty explaining the risk of COVID-19 infection to patients and convincing them to follow quarantine guidelines such as wearing masks and protective clothing, and performing COVID-19 tests [38, 39].

Affective symptoms of anxiety, such as fear, nervousness, and restlessness are associated with an increased risk of migraines, headaches, and muscle tension. In the current study, nurses have the highest median value on the hardiness aspect of the CD-RISC 25. According to Maramis and Cong [40], hardiness is a personality trait that has a defense function, so that when someone faces problems and workloads, they can do things that are considered appropriate to solve them. The results of this study indicate that nurses have low self-confidence, emotional regulation, and self-efficacy. A nurse needs to increase confidence in their abilities and control when facing a crisis or difficulty [41]. Characteristics such as self-control, expectations, and self-efficacy are significant contributors to nurse resilience [36, 41].

### 4.4. The Correlation between Work Stress and Anxiety, Resilience with Anxiety, and Work Stress with Resilience

These results support research in China and Turkey which shows that there is a significant relationship between work stress and anxiety; a positive value means that the higher the work stress, the higher the anxiety felt [13, 42]. Nurses who feel higher stress and anxiety will feel dissatisfied with their lives; besides that they experience physical and psychological exhaustion. Anxiety is an unpleasant emotional response to stress resulting from threats characterized by feelings of worry [43].

Pearson's correlation test showed no significant relationship between work stress and resilience. These results are different from those of the research by Hong et al. [9] in Korea. This happened because the nurses' work stress score in this study was below the average, while the research in Korea was conducted at the beginning of the pandemic, so it showed different results. Resilience is an adaptive coping strategy that can help reduce work stress and mental burden on health workers in response to treating patients infected with COVID-19 and preventing them from being infected with the virus during a pandemic [9].

Resilience is proven to have an important role in overcoming work pressure. In this study, there was no significant relationship between anxiety and resilience. According to studies conducted in Israel by Mosheva et al. [32], anxiety and resilience have a negative correlation. This implies that a nurse's resilience will be reduced the more anxious they are. This discrepancy in results came about because, in this study, most nurses did not experience anxiety.

## 5. Conclusions and Recommendations

This study provides information on the impact of COVID-19 on the psychosocial problems of psychiatric nurses. Nurses who provided nursing care to patients with mental disorders at the Central Mental Health Hospital during the COVID-19 pandemic had lower work stress scores than the average, experienced normal anxiety, and had moderate resilience scores. Work stress differs significantly depending on gender and contact frequency. Anxiety and contact frequency are significantly different, as are resilience and contact frequency. Work stress and anxiety have a considerable positive correlation. However, it should be noted that the sample was less diverse, and the number of participants was small. This study also presents the perspective of a psychiatric nurse.

We emphasize the findings in this study that psychiatric nurses need infection control guidance on infectious diseases such as COVID-19 inward service settings in mental health hospitals because patients and nurses have the right to safety and comfort from disease transmission. Also, when a pandemic occurs, the nurses do not need a long time to adapt to the illness, so the well-being of patients with mental disorders is not disturbed. Even though the results show moderate work stress and normal anxiety, they also need local support from ward leaders or hospital management in increasing their mental resilience and overcoming their psychosocial problems during a pandemic.

Future research should consider demographic variations, larger sample sizes, and government policies regarding infectious outbreak guidelines. Apart from that, the psychosocial problems of psychiatric nurses can also be further investigated when there is no pandemic.

## Figures and Tables

**Table 1:** Demographic characteristics (*n* = 101).

Demographics	Sample	Percentage (%)
Age		
19–40 years	44	43.6
41–65 years	57	56.4
Gender		
Male	33	32.7
Female	68	67.3
Marital status		
Married	97	96
Single/divorced	4	4
Education background		
Diploma	80	79.2
Bachelor	21	20.8
Working experience		
≤15 years	29	28.7
>15 years	72	71.3
Contact frequency		
≤5 times	43	42.6
>5 times	58	57.4

**Table 2:** Distribution levels of work stress, anxiety, and resilience of nurses (*n* = 101).

Variable	Mean ± SD	Median	Min–max	Sample	(%)
Work stress					
Score	45.1 ± 24.3				
Score lower than average				98	97
Score higher than average				3	3
Anxiety					
Score		26	20–39		
Normal/Not anxious				96	95.1
Mild-moderate				5	4.9
Severe				0	0
Panic				0	0
Resilience					
Score		81	69–100		
Low				29	28.7
Moderate				52	51.5
High				20	19.8

**Table 3:** Different tests of work stress, anxiety, and resilience based on the demographic characteristics of nurses (*n* = 101).

Demographics	Work stress			Anxiety		Resilience			
	Median (min–max)	Mean ± SD	*p*value	Median (min–max)	*p*value	Low (%)	Moderate (%)	High (%)	*p*value
Age									
19–40 years		45.77 ± 22.84	0.808^(a)^	26 (20–39)	0.205^(b)^	27	52.7	20.3	0.606^(d)^
41–65 years		44.58 ± 25.55		24 (20–39)		33.3	48.2	18.5	
Gender									
Male	29 (11–127)		0.013^∗(b)^	24 (20–39)	0.335^(b)^	15.2	66.6	18.2	0.229^(d)^
Female	47 (5–87)			26 (20–39)		35.3	44.1	20.6	
Marital status									
Married		45.54 ± 23.22	0.376^(a)^	26 (20–39)	0.847^(b)^	29.9	50.5	19.6	0.322^(e)^
Single/divorced		34.5 ± 27.33		25.5 (20–39)		0	75	25	
Education background									
Diploma		43.96 ± 23.17	0.361^(a)^	26 (20–39)	0.146^(b)^	30	48.7	21.3	0.964^(d)^
Bachelor		49.43 ± 28.39		23 (20–39)		23.8	61.9	14.3	
Work experience									
≤15 years		47.9 ± 23.82	0.465^(a)^	26 (20–39)	0.608^(b)^	31	55.2	13.8	0.444^(d)^
>15 years		43.97 ± 24.56		26 (20–39)		27.8	50	22.2	
Contact frequency									
≤5 times	53 (5–105)		0.002^*∗*(c)^	28 (20–39)	0.000^*∗*(b)^	34.9	58.1	7	0.019^*∗*(d)^
>5 times	61 (11–117)			24 (20–39)		24.1	46.6	29.3	

Notes: (a) independent *T*-test, (b) Mann–Whitney, (c) Kruskal–Wallis, (d) chi-square, and (e) Fischer test. ^*∗*^Significant (*p* < 0.05).

**Table 4:** Score on work stress, anxiety, and resilience of nurses based on domain on the ENSS, Z-SAS, and CD-RISC 25.

Domain	Mean ± SD	Median	Min–max
Work stress (ENSS)			
Death and dying	22.81 ± 12.19		0–60.71
Conflict with doctors	17.67 ± 12.64		0–60.00
Inadequate optional preparation	19.97 ± 14.44		0–58.33
Problems with peer support	13.2 ± 12.31		0–50.00
Problems with supervisors	16.55 ± 13.36		0–57.14
Uncertainty concerning treatment	25.91 ± 15.33		0–72.22
Problem with patients	24.07 ± 10.77		6.25–62.50
Workload	20.65 ± 11.96		0–63.89
Discrimination	4.29 ± 9.17		0–41.67
Anxiety (Z-SAS)			
Affective symptoms		6	5–10
Somatic symptoms		4	3–9
Cardiovascular system		2	2–5
Respiratory system		4	4–8
Gastrointerstinal system		1	1–3
Genitourinary system		1	1–3
Skin		3	2–6
Central nervous system		2	2–5
Resilience (CD-RISC 25)			
Hardiness		23	17–28
Coping		16	9–20
Adaptibility/flexibility		10	6–12
Meaningfulness/purpose		13	9–16
Optimism		6	3–6
Redulation of emotion and coping		6	4–8
Self-efficacy		6	6–8

**Table 5:** The correlation test between work stress, anxiety, and resilience in nurses.

Variables	Median (min–max)	Mean ± SD	*R*	*p* value
Work stress score		45.1 ± 24.3	0.299	0.002^*∗*(a)^
Anxiety score	26 (30–39)			

Anxiety score	26 (30–39)		0.47	0.643^(b)^
Resilience score	81 (69–100)			

Works stress score		45.1 ± 24.3	0.76	0.643^(a)^
Resilience score	81 (69–100)			

Notes: ^*∗*^Significant (*p* < 0.05); (a) Pearson's test, (b) Spearman's rank test; *r*, coefficient correlation.

## Data Availability

The authors declare that the data supporting the findings of this study are available within the article.
